# Overcoming hurdles: Enhancing post-mortem capabilities for neurological investigations in Africa

**DOI:** 10.1016/j.nbas.2023.100099

**Published:** 2023-09-28

**Authors:** Abdullahi Tunde Aborode, Ayah Karra-Aly, Seto Charles Ogunleye, Mercy Mayowa Olorunshola, Tayo Nafisat Folorunso, Christianah Oreoluwa Aloba, Adedayo Emmanuel Ogunware

**Affiliations:** a Healthy Africans Platform, Research and Development, Ibadan, Nigeria; b Schulich School of Medicine and Dentistry, University of Western Ontario, London, Ontario, Canada; c Department of Comparative Biomedical Sciences, College of Veterinary Medicine, Mississippi State University, 39760 MS, USA; d State University of New York at Binghamton, USA; e Department of Kinesiology, University of Louisiana at Lafayette, USA; f Department of Biomedical Sciences, University of Central Florida, USA; g Department of Neuroscience, Development and Regenerative Biology, The University of Texas at San Antonio, TX, USA

Post-mortem examinations, commonly called autopsies, are crucial for determining the incidence and understanding of neurological disorders [1]. There are several justifications for the significance of post-mortem investigations in this particular context. The post-mortem investigation provides one of the most definitive and precise diagnoses of neurological disorders [1]. Similar to several pathological conditions, certain neurological diseases exhibit symptom overlap or may manifest differently in each individual, making accurate diagnosis during their lifetime challenging. Post-mortem studies offer a valuable opportunity to comprehensively analyze the entire components of the nervous system, including the brain and spinal cord, detect any pathological alterations, and validate or modify the initial diagnosis from clinical examination, symptoms, and other diagnostic techniques [1].

The brevity of most neurological conditions with little or no identified clinical signs necessitates the identification of the underlying pathologies that are crucial in diagnoses, as they are characterized by distinct pathological alterations in the nervous system and brain [2]. Post-mortem investigations facilitate the identification of underlying pathological characteristics, including, but not limited to, abnormal protein aggregates (such as amyloid and neurofibrillary in Alzheimer's disease), neuronal loss, inflammation, or other structural abnormalities, by researchers and clinicians [2]. Understanding these fundamental pathologies is paramount in advancing our comprehension of disease mechanisms and pathogenesis and formulating precise and timely therapeutic interventions.

Research into the underlying mechanisms of neurological diseases facilitated by post-mortem studies offers invaluable opportunities [3], [4]. Scientists can comprehensively validate the physical signs alongside molecular, cellular, and genetic alterations linked to specific disorders by examining brain tissue samples; this contributes to elucidating essential mechanisms underlying disease onset and progression, as well as possible targets for therapeutic interventions, including the use of biomarkers and other biochemical vehicles. The process of validating biomarkers involves the assessment of measurable indicators that are utilized to detect or track the existence and advancement of medical conditions [4]. Post-mortem studies are of utmost importance in verifying and enhancing biomarkers for neurological disorders [5]. The comparison of biomarker levels identified during an individual's lifespan with post-mortem observations can facilitate the assessment of their precision and dependability, thereby enhancing the efficacy of diagnostic and monitoring instruments [5].

The efficacy of therapeutic interventions can be evaluated through post-mortem studies that analyze the brain tissue of individuals who have undergone specific treatments. The point above holds significant value in assessing the effects of various treatments on the pathological characteristics of neurological disorders and directing forthcoming therapeutic approaches [5], [6]. Genetic analysis can be conducted on post-mortem brain tissue to explore the involvement of particular genes in neurological disorders [6]. Comprehending the genetic elements that contribute to the vulnerability and advancement of diseases can offer valuable perspectives into implementing personalized medical strategies and potential intervention targets. Conducting post-mortem investigations is crucial for understanding neurological disorders [7]. They significantly contribute to precise diagnosis, identification of fundamental pathology, exploration of disease mechanisms, authentication of biomarkers, evaluation of treatment effectiveness, and genetic investigations [7]. The acquisition of this knowledge plays a crucial role in developing efficacious therapies, enhancing the quality of care provided to patients, and ultimately discovering remedies for neurological ailments.

As previously discussed, post-mortem studies are paramount in comprehending neurological disorders as they offer significant perspectives into the fundamentals of disease etiology and pathogenesis. Nonetheless, the execution of post-mortem investigations to effectively identify neurological disorders in many developing nations, such as nations of Africa, is impeded by various obstacles. Several African regions face constraints due to resources and infrastructure to conduct post-mortem examinations [8]. The availability and quality of facilities, such as laboratories with advanced equipment, proficient pathologists, and suitable storage for tissue samples, may be inadequate or insufficient [8].

Insufficient awareness and comprehension of the significance of post-mortem examinations for investigating neurological disorders could be observed among the general populace and healthcare practitioners [8]. This phenomenon may decrease the frequency of obtaining consent for post-mortem examinations, limiting the pool of available cases for scientific investigation. Cultural and religious beliefs can influence attitudes towards post-mortem examinations. Specific African communities may possess distinct cultural or religious customs that dissuade or forbid the conduct of autopsies, thereby presenting difficulties in securing familial authorization for post-mortem investigations.

Access to sophisticated diagnostic tools, such as molecular testing or imaging techniques, may be restricted in some areas of Africa due to limited availability and structural capabilities [9]. This phenomenon may impede the thorough examination of brain tissue and constrain the precision of identifying particular neurological disorders [9]. The continent of Africa presents many logistical and transportation obstacles due to its expansive size and varied topography [10]. Transporting brain tissue samples to specialized research centers or laboratories for detailed analysis can present challenges, especially in geographically isolated regions with limited transportation infrastructure.

Proper documentation and data collection are indeed essential components of post-mortem investigations. They help provide context to the pathological findings and serve as a reference for future research and comparisons. However, certain African regions may face challenges in these areas due to inadequate or fragmented medical records and data collection systems [11]. The absence of accurate clinical histories and findings can significantly affect the quality and interpretability of post-mortem studies, limiting their utility in elucidating the underlying mechanisms of neurological disorders. Other logistical and infrastructural issues, including limited access to technological tools for data recording and storage, a lack of standardized procedures for medical record-keeping, and insufficient training in data management, compound the problem of inconsistent or inadequate documentation. Additionally, the high patient-to-doctor ratio in many African regions [12] may contribute to the difficulties in maintaining comprehensive and up-to-date patient records.

Ethical considerations are another vital aspect of post-mortem studies [13]. It is crucial to ensure that these studies are conducted with the utmost respect for the deceased and their families, taking into account cultural and religious sensitivities. This includes obtaining informed consent, ensuring privacy and confidentiality of patient information, and respecting cultural practices and beliefs surrounding death and autopsy.

Informed consent, in particular, is a key requirement for post-mortem studies. It is essential to ensure that families understand the purpose of the autopsy, the procedures involved, and how the findings could contribute to medical knowledge and patient care. All of these should be communicated clearly and sensitively in a manner that respects the grieving process [14]. Furthermore, privacy and confidentiality must be upheld in all post-mortem studies [15]. This involves protecting the identity of the deceased and their families and ensuring that the findings are reported in a manner that does not disclose any identifying information. Lastly, cultural sensitivity is crucial in the ethical conduct of post-mortem studies [16]. It is essential to respect the cultural and religious beliefs of the deceased and their families and consider these when planning and conducting autopsies. This might involve adapting the procedures to accommodate cultural practices or consulting with community leaders to ensure cultural appropriateness.

Overcoming the challenges posed by conducting post-mortem investigations in Africa requires a concerted effort among all stakeholders, including healthcare professionals, researchers, policymakers, and society. Such a collaborative approach could significantly enhance the continent's understanding and management of neurological disorders. To begin with, resources must be allocated strategically to strengthen the healthcare infrastructure, which forms the backbone of medical research and treatment. This could involve improving laboratory facilities, enhancing storage capabilities for biological samples, and investing in advanced diagnostic technologies. By developing the necessary infrastructure, we could increase the accuracy of post-mortem studies and facilitate more comprehensive research into neurological disorders.

Education also plays a pivotal role in addressing the challenges related to post-mortem studies. This could involve educating the general public about the significance of these investigations, which will foster a more positive attitude towards them and potentially increase the number of people consenting to post-mortem examinations. Public awareness initiatives are equally essential. These initiatives could focus on disseminating information about neurological disorders and the value of post-mortem studies in advancing medical knowledge and improving patient care. By raising public awareness, we could address some of the cultural and religious concerns related to autopsies, thereby increasing societal acceptance and support for these studies.

Lastly, collaborations with international entities and academic institutions can help overcome some of the unique challenges associated with post-mortem studies in Africa [17]. Such partnerships enable sharing of expertise, resources, and knowledge, which is invaluable in navigating the obstacles to conducting post-mortem examinations in Africa. This could involve exchange programs for researchers and healthcare professionals, joint research projects, and access to advanced research facilities and technologies.

In order to obtain a globally comprehensive understanding of the etiopathogenesis, course, and treatment responses of neurological diseases, it is imperative to adopt a standardized approach to collecting autopsy data, a method that would necessitate the formulation of universally accepted guidelines and protocols that take into consideration not only the diverse clinical presentations across regions but also the unique genetic, environmental, and sociocultural factors inherent to each location, particularly emphasizing regions like Africa, which has traditionally been underrepresented in neuropathological research; by encouraging collaboration between national and international bodies, fostering training and capacity-building initiatives, leveraging technological advancements for seamless data collection and integration [18], and ensuring stringent quality control, it becomes possible to create a vast and invaluable research database that can be interrogated and analyzed in a manner that brings forth meaningful comparisons and insights into the neurological diseases prevalent in Africa and across the globe, and, most crucially, such an initiative would not only serve a genuine research purpose by filling existing knowledge gaps but also amplify Africa's role and visibility within the international neuropathology research community, propelling further scientific investigations and collaborations.

## Declaration of Competing Interest

The authors declare that they have no known competing financial interests or personal relationships that could have appeared to influence the work reported in this paper.
